# Exploring the experiences of content experts with item vetting during item bank development

**DOI:** 10.12669/pjms.40.6.8664

**Published:** 2024-07

**Authors:** Anbreen Aziz, Mashaal Sabqat, Faiza Kiran, Tayyeba Iftikhar Mirza

**Affiliations:** 1 Anbreen Aziz, BDS, MHPE, AFHEA Assistant Professor, Department of Medical Education, HBS Medical and Dental College, Islamabad; 2 Mashaal Sabqat, MBBS, MHPE Assistant Professor, Medical Education, Assistant Director Riphah Institute of Assessment, Riphah International University, Islamabad; 3 Faiza Kiran, MBBS, MHPE Assistant Professor, Department of Medical Education, Shifa School of Health Professions Education, Shifa Tameer e Millat University, Islamabad, Pakistan; 4 Tayyeba Iftikhar Mirza, MBBS, MHPE Assistant Professor, Department of Medical Education, Foundation University Medical College, Islamabad

**Keywords:** Examination questions, Quality assessments, Medical education

## Abstract

**Objective:**

To explore content experts’ experiences with item vetting during item bank development at a public sector medical university of Rawalpindi, Pakistan.

**Methods:**

An exploratory study was carried out from December 2022 to February 2023 at a public sector medical college of Rawalpindi. A purposive sampling technique was employed to collect data from all content experts of the study institute who participated in item vetting activity during pre-exam moderation in the university. A pilot-tested semi-structured interview guide was utilized, interviews were audio recorded and later transcribed. Participants’ anonymity was ensured. Various quality assurance strategies were employed to ensure the trustworthiness of the findings. Thematic analysis was performed on the transcribed data and themes were finalized by achieving consensus among all authors.

**Results:**

Six themes overarching the fourteen subthemes emerged from the data. Participants expressed a profound sense of satisfaction and valued their experience in refining expertise in constructing multiple-choice questions (MCQs). It was widely acknowledged that such activities not only contribute to the enhancement of item development skills but also improve quality of items.

**Conclusions:**

The consistent implementation of item vetting routines, in conjunction with diligent adherence to item writing protocols, contributes to quality assurance measures in assessment. Item bank development for fair and transparent assessment ensures production of competent healthcare professionals filtering incompetent ones hence improving health care services in the community.

## INTRODUCTION

Valid assessment in a medical school ensures the production of competent doctors.^1^ Knowledge, skills and attitudes gained by medical students are evaluated by a variety of assessment methods.^1^ MCQs are written assessment methods frequently used to test higher order thinking skills.^2^ They carry advantages of testing a wide range of content, assessing a large set of examinees and a rapid scoring system.^2^,^3^ They are proved to be valid and reliable if constructed properly.^2^ A good quality MCQ consists of a detailed clinical scenario-based statement, a clearly asked question in the form of a lead-in and one most appropriate answer along with three or four functional distractors.^2^ Once an MCQ is constructed according to the recommended guidelines, it should be reviewed by the vetting committee which consists of an item author who must be a content expert, along with a medical education expert to review it from various aspects before testing in exams.^1^,^4^-^6^ Vetting is a pre-test review and evaluation of items; items are corrected by detecting item writing flaws, checking content and grammatical/language errors to improve their quality.^4^,^7^ The psychometric properties of these vetted items are improved post-test, hence lead to development of item bank.^4^

Though literature is replete with studies signifying faculty training and item vetting in improving the quality of MCQs, many medical institutes do not employ item vetting strategy. Moreover, in Pakistani context, limited studies are available on the impact of item vetting towards improvement in the quality of MCQs. One of the reasons could be that the item vetting is not being carried out in most of the medical institutes of Pakistan. Therefore, the items used within medical colleges are often poorly constructed having flaws in them.^2^

A previous study from Pakistan exploring the barriers and facilitators in writing good quality MCQs recommends obtaining faculty perceptions after conducting workshops on item construction and performing item vetting.^8^ Therefore, our study aimed to explore the perceptions of faculty regarding their item vetting experiences. This exploration holds significance in our context as it serves as a needs assessment for designing related faculty development initiatives, based on faculty input. Such thoughtfully designed faculty development programs will help enhance faculty competence in producing valid and reliable items.^9^ This in turn, will lead to a fair assessment of students’ abilities, ultimately leading towards the production of competent physicians, and improving health care services in the community.

## METHODS

A qualitative exploratory study was conducted in a public sector medical college of Rawalpindi from December 2022 - February 2023.

### Ethical Approval:

It was obtained by the ethics review committee of Army Medical College (ERC/ ID/ 244, Dated: December 12, 2022).

### Participants and procedure:

Purposive sampling was used to gain an in-depth understanding of the participants’ perspectives. All content experts (Assistant Professor and above) of the study institute who were involved in item vetting activity during pre-exam moderation were invited to participate in the study via an email comprising a brief introduction of the study. In-depth, semi-structured interviews ranging from 30 to 40 minutes were conducted and recorded by AA, MS and TIM on phone, till data saturation, which was achieved at the thirteenth participant. The interviews were transcribed by MS and TIM.

### Interview guide development:

Comprehensive literature review was done by AA and the interview guide was developed based on the derived themes by discussion among all authors. The interview questions were validated by two medical educationalists, and pilot-tested with two subject experts.

### Data Analysis:

As the data constituted less than 500 pages, manual thematic analysis was performed,^10^ and themes and subthemes were extracted by highlighting key features, similarities, and differences in opinions of participants, and generating collaborative insights amongst the authors.

### Quality assurance strategies:

The authors used the quality assurance criteria for naturalistic studies by Lincoln and Guba for this study.^11^ The strategies used for quality assurance included three peer debriefing sessions,^12^ maintaining a reflective journal to note down the non-verbal cues of the participants during interviews,^13^ and sending the interview transcripts to the respective participants for member checking to ensure credibility.^13^ Moreover, data analysis was done by two authors, and the analysis was verified with all authors to ensure data analysis triangulation.

## RESULTS

A total of thirteen faculty members participated in the study: seven from basic sciences (53.84%) and six from clinical sciences (46.15%). These participants took part in a two-month item vetting activity, held in a public sector medical university from mid-July to mid-September 2022. This activity was held in the form of teams and each team comprised of three subject experts and one medical education expert from the affiliated colleges. The vetted items were planned to be part of the final professional examination of the university. The university granted remuneration to the team members. Data analysis generated fourteen sub-themes that were conjured into six themes. (Table-I)

**Table-I:** Themes, sub-themes and corresponding quotes generated by data analysis.

Subthemes	Codes	Participant’s quotations
***Theme-1: Organized and Fruitful Activity***		
Systematic process	Systematic with both departments and university involvement Methodical & organized process	It is a good activity…because it is running now systematically at departmental as well as university level. Previously things were going haphazardly without any feedback and changes at university level. In my department I did it for many years, but now the whole process is more organized. (R5)
MCQ standardization	Useful due to standardization	A criterion has been set and everyone has to follow that. This helps in standardizing the MCQs. Everyone has to make MCQs of the same standard. (R1)
***Theme-2: Meaningful Experiential Learning***		
Hands-on/ Practical experience	Hands-on activity Practical skills-based activity	I learned by doing hands-on activity and gained skill of moderating MCQs; previously it was theoretical with no practical experience. (R15)
Input of multiple minds (Diverse faculty with educationalist)	Useful because diverse faculty and educationalists involved Interaction with other institutions’ faculty	When you have more people in a team, they point out some sort of confusion in MCQ… then one realizes that students can also think along the same lines, then you modify the question accordingly. This has helped me a lot in making the stem of the MCQ crisper and clearer. (R7) It was a very useful activity because, at times, we are unable to see the flaws while constructing the MCQ but when we look at it from the perspective of multiple minds, things get better. (R2)
Multiple subject experts to remove bias	Multiple subject experts to remove bias Experts coming together Expert consensus	I like the overall activity for engaging multiple specialists from different medical colleges. This helps to remove bias. (R10)
***Theme-3: Increased Confidence***		
Interaction with other college’s faculty	Interaction with faculty from other institutions	They invited faculty of top minds from different institutes affiliated with this university. I was happy that we will be on the same page from now onwards. One regains confidence by interacting with faculty from other institutes (R11)
***Theme-4: Enhanced Test/ Item Writing Skills***		
Awareness about item writing flaws	Learned about alignment between stem & lead-in Learned about technical mistakes in stem, lead-in and options Learned about grammatical errors Learned about cover uncover test	Many times, through the help of the vetting team, I was able to replace the nonfunctional distractors with better options, which never came to my mind while making the MCQs. (R2) All types of flaws related to the stem and in scenario making, and asking lead in the form of question are improved. (R5) Most of the mistakes in my MCQs were grammatical errors. I found out that the stem and lead-in should be aligned. I remember we used to put the stem for the sake of having a scenario on the top; it had no relation with the lead-in. The options were never aligned; we would just put the options of our own choice. (R4) The cover uncover test was very new for me. I had never thought that each component of MCQ should be aligned. (R1)
Development of application-level items for basic sciences	Difficult to make application level MCQs for basic science subjects Learned about difficulty level of MCQs	It is hard to make application level MCQs/ scenarios in basic sciences subjects when curriculum is traditional, and students are not taught with clinical relevance. (R13) Most common flaws in MCQs were … too easy options which lower the difficulty index of item. A little hard work can improve the difficulty index and hence the quality of MCQ. (R14)
Applied the knowledge gained during certificate of health professions education (CHPE)	Putting CHPE knowledge to practice Skills were polished due to practice Practical experience on top of basic knowledge	I had learned the theoretical part of MCQ construction in my CHPE course, and when I tried applying that knowledge even then I was making mistakes. When you have hands-on experience, you learn many things. (R3)
***Theme-5: Impact on Quality Assurance in Assessment***		
Improved quantity and quality of MCQs	Improved quality of bank Improved quantity of MCQs Useful due to item bank development	The quantity of good constructed MCQs definitely has been increased after this activity. (R5) To become supervisor, I need to make many MCQs, and I think this exercise has helped me in learning how to make them. (R14) My skills are polished due to this item bank activity. I can now make good quality MCQs. I believe practice and repetition of the activity make a man perfect. (R7)
Improved psychometric properties i.e., improved clarity of MCQs	Improved clarity of MCQs Improved cognitive level of MCQs Better reliability and validity of MCQs Positive impact on content validity of exam	These questions will be given in exams and for the students it will be easier to understand the questions. Previously, some questions had no clarity, options had double meanings, now they are crystal clear. (R15) Regarding item vetting activity … lets us review, recheck and re-evaluate; overall it improves the questions for all types of learners. Obviously, you can make it more reliable and valid. (R2) Most of the Table of Specification (TOS) was ignored previously while making paper. This activity will be helpful in future while making papers from vetted pool. (R15) I believe when the vetted MCQs are given to the students, they will study deeply, as the majority are problem-solving, and students will have to read their topics thoroughly in order to attempt these MCQs. (R3)
	Deep learning promoted due to improved quality MCQs	Of course, the assessment quality will improve. Students will have a deep approach rather than rote learning.(R8)
	Positive impact on student learning due to application level MCQs	…students will learn extensively as paper will come from all topics. The will also study applied aspects of basic sciences as clinical scenarios are added now. (R15)
***Theme-6: Faculty Recommendations***		
Fulfilling faculty needs	Due credit should be given to faculty involved. Faculty members should be informed in advance. Should be less strenuous for faculty Workshop on MCQs and post hoc analysis needed	Faculty who participates in this activity must be given an incentive as it is a time-consuming participation. (R5) The faculty members should be informed about the activity well in advance so that they can be prepared and also manage their time with their workload. (R14) There must be a uniform distribution of workload by involving other members of the department, provided they are trained for this purpose. (R5) There should be a certain count to be vetted in a go, or in a setting because tired minds cannot give good performance. (R6) There should be faculty development workshops in which they should be taught, how to construct MCQs and then the faculty should be given the task to bring the MCQs that they have constructed or modified after the workshop so that they can be vetted by experts. (R9) I think I want to attend workshops on MCQs and post-hoc analysis to improve myself. (R13) There should be interdepartmental meetings which should discuss a few MCQs weekly, or bimonthly but regularly. After those meetings, you can take the vetted MCQs to the medical educationist to further assess the different aspects of a good MCQ. Finally, they can be vetted again in a centralized manner within university. Then it can be a more fruitful and productive activity. (R2)
Frequency of item vetting activity	Should be regular but at intervals to accommodate faculty workload (Frequency: once a year Frequency: at least twice a year Frequency: 4 times a year) Progressive item vetting at different levels	It should be carried out twice a year if not more than that. (R3) Whatever I have learned through this activity. Such activities should be held on a regular basis and each faculty of the department should be involved in such activities. (R8)

*R= respondent.

### Organized and Fruitful Activity with Meaningful Learning Experience:

All participants found the opportunity to interact with subject experts and medical educationalists from diverse institutions, hence had valuable learning experience. They appreciated the systematic and organized process and expressed that the knowledge gained during these days was considerably more practical and useful. Many participants reported that they have learned about technical and grammatical errors, others learned about cover-uncover test and alignment between lead-in and options. In particular, basic sciences faculty noted a significant improvement in their ability to create application-level MCQs, which they previously found challenging.

### Impact on Quality Assurance in Assessment:

According to the participants, the activity increased the number of reviewed items, facilitating the development of item bank. The activity also improved content validity by segregating the items according to the table of specifications and highlighted many important learning objectives that were not being assessed previously.

### Recommendations:

The participants provided several recommendations for improving the item vetting process. They suggested giving due credit to the faculty members in the form of remuneration to motivate them, planning strategically, informing the faculty members in advance to enable them to manage their time well, and distributing the workload uniformly among faculty members to prevent exhaustion. Finally, the participants suggested a stepwise approach to item vetting: initial scrutiny and improvement of items in departmental meetings, then assessment by medical educationalist, and finally, a central vetting process for enhanced efficiency and effectiveness.

## DISCUSSION

The aim of this study was to explore the perspectives of faculty members involved in the item vetting process at a university. We found that a well-organized item vetting activity has a positive impact on both the quality of MCQs and the learning of the faculty regarding MCQ construction and its flaws. The common item flaws observed in this study were technical, such as longest option being correct, convergence, and negatively phrased lead-in. Furthermore, unfocused stems, and the use of excessive verbiage were noted in item construction, as also discussed in Tarrant and Ware’s study.^14^ Downing has explained that such errors are made mostly by novice item writers who are aware of item writing guidelines but fail to adhere to them.^15^

The current study confirmed Downing’s reasoning as it was found that despite their prior participation in several workshops on MCQ construction, the MCQs submitted by faculty had multiple item flaws. The reason for the lack of a discernable impact of the aforementioned workshops could be a lack of experiential learning, as shown by the current study’s results. Faculty members expressed appreciation for experiential learning facilitated by educationalists during the process. This aligns with the findings of Kiran^16^ and Notzer^17^, who emphasized the transformational nature of learning during hands-on practice and feedback in skill enhancement.

Additionally, faculty members acknowledged their increased confidence in MCQ construction after this activity, as also highlighted in a study conducted by Al Faris et al.^18^ Considering the two-month duration of the activity and its discernable impact on the participant’s perceptions, our results mirrored those of a study by Iramaneerat, confirming that sessions on MCQ writing and analysis, regardless of their duration, enhance faculty members’ test-writing skills.^19^ We propose the implementation of more frequent, well-planned, and organized item vetting activities involving both junior and senior faculty members. Although this activity was demanding in terms of time, commitment, availability, and transportation logistics, faculty members found it more favorable as it provided a unique, albeit exhausting, yet meaningful learning experience.

This study has shown that item vetting in medical education improves item writing skills and satisfaction of faculty, transforms their perspective on item construction, improves content validity, and enhances quality assurance in assessment. As assessment drives learning, high quality MCQs assessing application of knowledge will impact students’ learning style and promote deep learning in medical students. This, eventually, will have a positive effect on healthcare system by producing competent healthcare professionals, and filtering incompetent ones by robust assessment.

### Strengths of the study:

This study has highlighted the significance of incorporating quality assurance procedures in assessment.

### Limitations:

The subject experts from all affiliated medical colleges should have been included in the study.

## CONCLUSION

Regular item vetting exercises, accompanied by adherence to item writing guidelines, are widely embraced by faculty members due to their efficacy in yielding positive learning outcomes and improving content validity of assessment. This is primarily attributed to the inclusion of hands-on practice and timely provision of constructive feedback within the vetting process. Recommendations provided by the faculty should be helpful in future activities. Moreover, other universities can follow the same pattern of item bank development for fair and transparent assessment.

### Authors Contribution:

**AA:** Conceived the idea and made a proposal.

**MS** and **TIM:** Collected the data.

**AA, MS, FK** and **TIM:** Did data analysis and interpretation.

**AA, MS** and **FK:** Drafted manuscript.

All authors finalized the manuscript before submission and are accountable for the integrity of research.
